# Histopathological Patterns of Metastatic Cutaneous Squamous Cell Carcinoma to Intra‐Parotid Lymph Nodes and Correlation With Outcomes

**DOI:** 10.1002/oto2.70284

**Published:** 2026-09-25

**Authors:** Mayuri Yasuda, Aayushma Regmi, Maitri Mehta, Cullen M. Lilley, Eric Thorpe, Swati Mehrotra, Andrea Ziegler, Vijayalakshmi Ananthanarayanan

**Affiliations:** ^1^ Department of Otolaryngology–Head and Neck Surgery Loyola University Medical Center Maywood Illinois USA; ^2^ Department of Pathology Loyola University Medical Center Maywood Illinois USA; ^3^ Present address: Tareen Dermatology Roseville Minnesota USA; ^4^ Present address: Department of Laboratory Medicine and Pathology, Mayo Clinic Rochester Minnesota USA; ^5^ Present address: Department of Pathology and Laboratory Medicine University of California Los Angeles Los Angeles California USA; ^6^ Present address: Department of Pathology Edward Hines Jr., Veterans Administration Hospital Hines Illinois USA; ^7^ Present address: Department of Pathology University of Illinois at Chicago Chicago Illinois USA

**Keywords:** lymph node metastases, parotid, perineural invasion, squamous cell carcinoma, worst pattern of invasion

## Abstract

**Objective:**

Metastatic cutaneous squamous cell carcinoma (cSCC) to the parotid gland is an uncommon but serious disease process. This study evaluates the histopathological patterns of cSCC metastases to the parotid and correlates with recurrence rates and other clinical outcomes.

**Study Design:**

This was a retrospective cohort study.

**Setting:**

The study was conducted at a single tertiary care center.

**Methods:**

Cases of cSCC with metastasis to the parotid between 2016 and 2022 were included. The histological slides from the parotidectomy were reviewed to determine the WPOI. The patient's history, operative and surgical pathology reports, and postoperative clinic notes were also reviewed.

**Results:**

There was a total of 46 primary tumors. Most cases were located on the pinna, followed by temple and scalp. Patients with WPOI 4/5 were more likely to have received prior adjuvant treatment after initial excision of their primary (*P* = .035).

These tumors were clinically aggressive. 54.3% had perineural invasion (PNI). Histologically, 71.1% were infiltrative with a median WPOI of 3. PNI was the only statistically significant histological factor associated with a higher risk of postparotidectomy recurrence (*P* = .036). Univariate logistic regression showed that patients with PNI were more likely to have recurrence (OR 8.33, *P* = .023).

**Conclusion:**

There were no statistically significant differences in outcomes when comparing WPOI groups. However, PNI, even at a metastatic site, was noted to be a significant risk factor for recurrence in these tumors, supporting prior evidence that PNI is an important factor to consider when determining treatment options in these clinically aggressive malignancies.

Primary cutaneous squamous cell carcinoma (cSCC) is the second most common malignancy in humans after basal cell carcinoma.^1^ The head and neck region is the most common origin site for cSCC, accounting for 80% to 90% of cases.^2^ It is commonly associated with ultraviolet light exposure.

Most patients do very well after diagnosis, however, metastasis has been reported in 1.5% to 5.2% of patients.^3^, ^4^ Metastasis typically occurs in the regional lymph node basin.^4^ The development of regional lymph node metastasis decreases survival rate significantly. Five‐year survival rates in this subset of patients range between 50% and 70%.^5^

Metastasis specifically to the parotid gland is an uncommon but adverse manifestation of the disease process. It has been shown to occur in only 1% to 3% of patients with head and neck cSCC.^6^ It commonly presents as a parotid mass mimicking a primary salivary gland tumor. Fine needle aspiration is often performed to obtain a diagnosis. Following diagnosis, surgical management includes parotidectomy with or without neck dissection for optimal locoregional disease control. This is often followed by adjuvant radiation therapy.

While clinical management protocols for parotid metastasis of primary cSCC have been well‐described, there has yet to be a study to describe the histopathological patterns of metastasis to the parotid. Specifically, worst pattern of invasion (WPOI) is a histopathologic factor that has been recently highlighted as a poor prognosticator in oral cavity SCC^7^. WPOI as a prognostication tool in cSCC of the head and neck region, on the other hand, has not been extensively studied.

WPOI is an established adverse histopathologic feature in mucosal squamous cell carcinoma of the head and neck, but its prognostic significance in head and neck cSCC remains incompletely characterized. Agar et al identified small tumor islands and tumor satellites as independent adverse predictors of nodal metastasis in cSCC, supporting the broader concept that the architecture of tumor invasion may reflect clinically relevant biologic behavior.^8^

At tertiary referral centers, review of the primary cutaneous tumor is often limited by the fact that the initial biopsy or excision is performed in outpatient or community practice settings and may not be available for centralized pathologic assessment. In such cases, metastatic tissue may represent the only available substrate for morphologic risk stratification. The use of metastatic site histomorphology for prognostic assessment is biologically plausible and has precedent in other solid tumors. For example, histopathologic growth patterns of liver metastases from colorectal carcinoma and melanoma, including replacement and desmoplastic growth patterns, have been associated with patient outcomes.^9^, ^10^

We therefore hypothesized that the pattern of invasion at the metastatic site, specifically within parotid metastases from head and neck cSCC, may provide clinically meaningful prognostic information. We further hypothesized that tumors with more aggressive biology would show infiltrative metastatic growth patterns, such as single‐cell invasion, dispersed tumor growth, or small infiltrative nests analogous to WPOI‐4/5, whereas tumors with less aggressive behavior would more often display pushing, expansile, or cohesive nested growth. This framework treats the metastatic tumor‐host interface as a surrogate morphologic readout of tumor aggressiveness in cases where primary‐site pathologic material is unavailable.

## Methods

This study was a retrospective chart review of patients at a single tertiary care center. IRB exemption was granted by the Loyola Institutional Review Board. Cases of primary cSCC metastatic to the parotid gland diagnosed during 2016 to 2022 were retrieved retrospectively from pathology department archives. Cases with direct involvement of the parotid gland from the primary tumor were excluded. Pathologic slides from the parotidectomy from each case were reviewed and analyzed by 2 qualified individuals with strong interrater reliability. The index primary cutaneous tumors were not available for comparison or evaluation in any of the cases. All histopathological and clinical data were compiled and analyzed.

**Figure 1:**
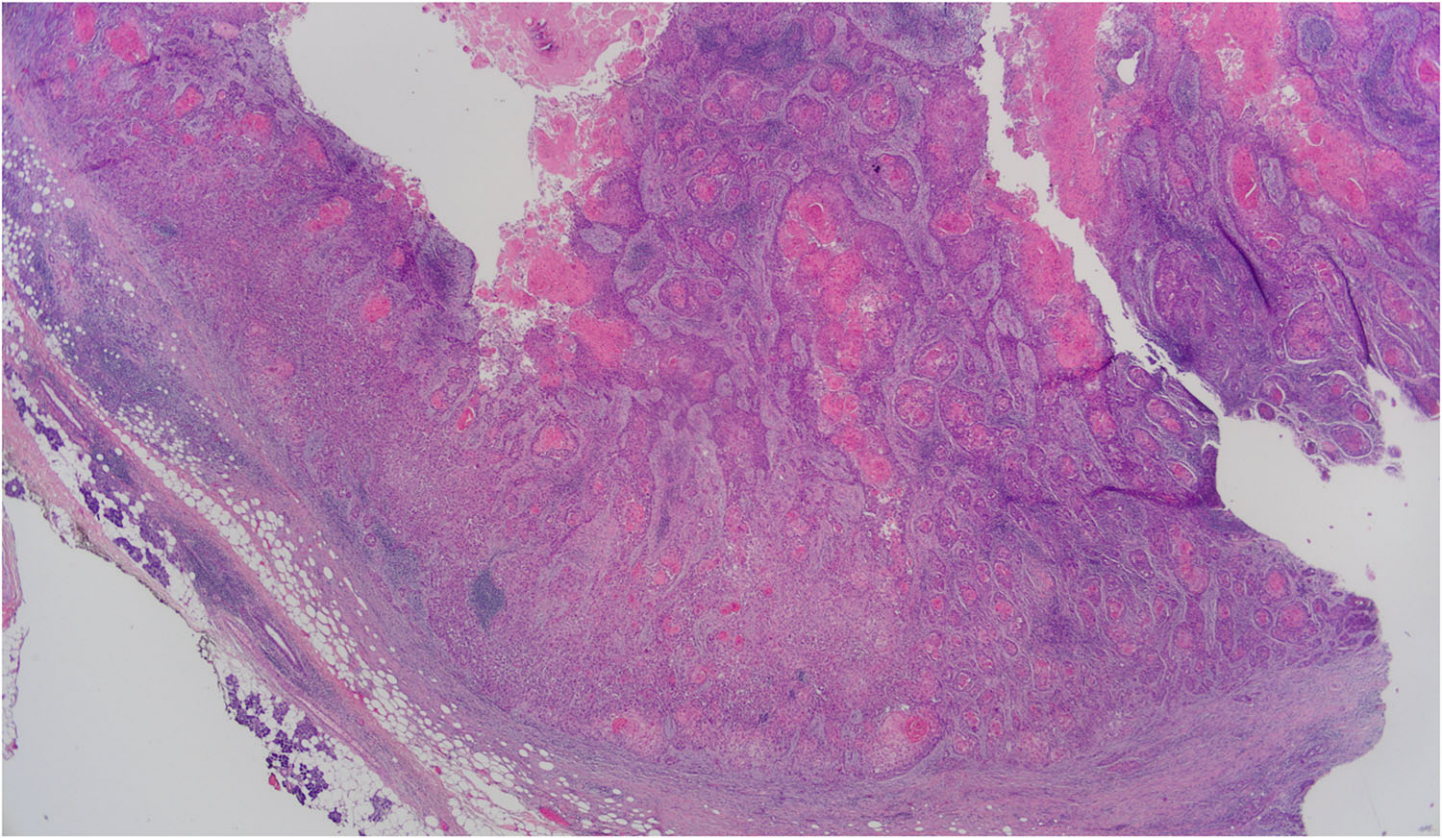
WPOI 2‐3 (WPOI 2 [infiltrating solid cords, bands, and/or strands], WPOI 3 [small groups or cords of infiltrating cells, n > 15]) with normal salivary gland tissue at the periphery.

**Figure 2:**
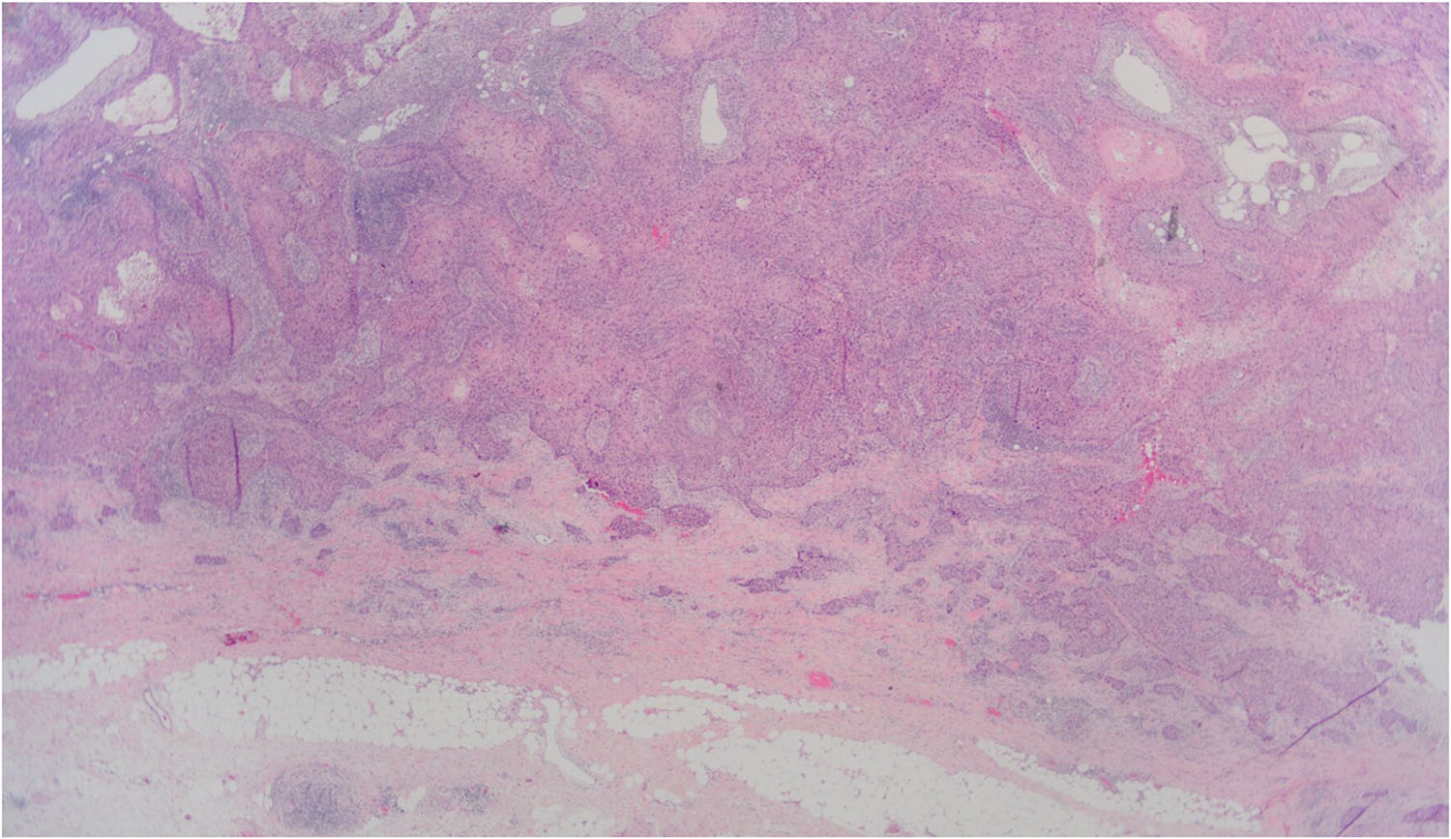
WPOI 4 (marked and widespread cellular dissociation in small groups of cells, n < 15, and/or in single cells).

The histological slides were reviewed to determine the worst pattern of invasion (WPOI). These categories included WPOI 1 (pushing, well‐delineated infiltrating borders), WPOI 2 (infiltrating solid cords, bands, and/or strands), WPOI 3 (small groups or cords of infiltrating cells, n > 15) (Figure 1), WPOI 4 (marked and widespread cellular dissociation in small groups of cells, n < 15, and/or in single cells) (Figure 2), and WPOI 5 (tumor satellites of any size located ≥1 mm away from the main tumor or the next closest satellite with intervening normal tissue).^11^, ^12^, ^13^, ^14^

Statistical analysis was performed using IBM SPSS Statistics, Version 29. Chi‐squared testing was used for categorical variables and two‐tailed *t*‐test for continuous variables between WPOI groups and in patients with and without recurrence. Univariate logistic regression was performed for patients with any type of recurrence compared to those without recurrence. Multivariate analysis was not performed due to limitations of the small study size.

## Results

There was a total of 46 cases. There were 41 males and 5 females. The median age was 76.5 years old (range: 59‐95 years old). Twenty patients had a history of tobacco use (43.5%). Eight patients were immunocompromised (17.4%). Most primary tumors were located on the pinna (48%), followed by temple (28%), and scalp (11%) (Table 1).

**Table 1:** Demographics, Treatment, and Recurrence Information in Patients Stratified by WPOI

	Total	WPOI 1‐3	WPOI 4‐5	*P*‐value
Number	46	26 (56.5%)	20 (43.5%)	
Gender (male)	41 (89.1%)	24 (92.3%)	17 (85.0%)	.43
Age (years)	76.5 (59‐95)	76.6 (59‐91)	76.3 (59‐95)	.91
Smoking history	20 (43.5%)	11 (42.3%)	9 (45.0%)	.86
Immune compromised	8 (17.4%)	5 (19.2%)	3 (15.0%)	.71
Primary site				
Ear/periauricular	22 (47.8%)	17 (65.4%)	5 (25.0%)	.0066
Scalp/forehead	5 (10.9%)	2 (7.7%)	3 (15.0%)	.43
Temple/cheek	13 (28.2%)	5 (19.2%)	8 (40.0%)	.12
Other	3 (6.5%)	1 (3.8%)	2 (10.0%)	.40
Unknown primary site	3 (6.5%)	1 (3.8%)	2 (10.0%)	.40
Prior treatment				
None	18 (39.1%)	10 (38.5%)	8 (40.0%)	.92
Mohs/excision	21 (45.7%)	15 (57.7%)	6 (30.0%)	.062
Excision + Adjuvant RT/CRT	6 (13.0%)	1 (3.8%)	5 (25.0%)	.035
CRT	1 (2.2%)	0	1 (5.0%)	.25
Adjuvant treatment				
None	13 (28.3%)	6 (23.1%)	7 (35.0%)	.37
RT	18 (39.1%)	12 (46.1%)	6 (30.0%)	.27
CRT	8 (17.4%)	2 (7.7%)	6 (30.0%)	.048
Unknown	7 (15.2%)	6 (23.1%)	1 (5.0%)	.090
Recurrence	11 (23.9%)	6 (23.1%)	5 (25.0%)	.88
Local	9 (19.6%)	4 (15.4%)	5 (25.0%)	.42
Regional	1 (2.2%)	1 (3.8%)	0	.38
Distant	2 (4.3%)	2 (7.7%)	0	.20

Abbreviations: CRT, concurrent chemotherapy and radiation therapy; RT, radiation therapy; WPOI, worst pattern of invasion.

As expected, compared to patients with WPOI 1‐3, patients with WPOI 4/5 were more likely to have had a prior excision with adjuvant treatment (chemoradiation and/or radiation) (*P* = .035). Similarly, patients with WPOI 4/5 invasion pattern at the metastatic sites were more likely to receive adjuvant treatment with chemotherapy and radiation after surgical excision of the parotid (*P* = .048). There were no significant differences between the WPOI groups in terms of recurrence (Table 1).

When comparing patients who had a recurrence to those who did not, there were no significant demographic differences (Table 2).

**Table 2:** Demographics, Treatment, and Recurrence Information in Patients Stratified by Recurrence

	Total	Recurrence	No recurrence	*P*‐value
Number	46	11 (23.9%)	35 (76.1%)	
Gender (male)	41 (89.1%)	11 (100%)	30 (85.7%)	.18
Age (years)	76.5 (59‐95)	78.7 (72‐95)	75.8 (59‐91)	.35
Smoking history	20 (43.5%)	7 (63.6%)	13 (37.1%)	.12
Immune compromised	8 (17.4%)	3 (27.2%)	5 (14.3%)	.32
Primary site				
Ear/periauricular	22 (47.8%)	4 (36.3%)	18 (51.4%)	.38
Scalp/forehead	5 (10.9%)	2 (18.2%)	3 (8.6%)	.37
Temple/cheek	13 (28.3%)	5 (45.5%)	8 (22.9%)	.15
Other	3 (6.5%)	0	3 (8.6%)	.32
Unknown primary site	3 (6.5%)	0	3 (8.6%)	.32
Prior treatment				
None	18 (39.1%)	2 (18.2%)	16 (45.7%)	.10
Mohs/excision	21 (45.6%)	5 (45.5%)	16 (45.7%)	.99
Excision + Adjuvant RT/CRT	6 (13.0%)	3 (27.2%)	3 (8.6%)	.11
CRT	1 (2.2%)	1 (9.1%)	0	.071
Adjuvant Treatment				
None	13 (28.3%)	3 (27.2%)	10 (28.6%)	.93
RT	18 (39.1%)	5 (45.5%)	13 (37.1%)	.62
CRT	8 (17.4%)	2 (18.2%)	6 (17.1%)	.94
Unknown	7 (15.2%)	1 (9.1%)	6 (17.1%)	.52

Abbreviations: CRT, concurrent chemotherapy and radiation therapy; RT, radiation therapy.

As shown in Table 3, the parotid gland tumors or metastases to the parotid gland demonstrated aggressive pathological features. Half of the patients had tumors classified as 20% to 50% keratinizing squamous cell carcinoma (SCC). The most common histologic pattern observed was pseudoglandular/acantholytic (43.9%), followed by diffuse sheet‐like growth (18.1%). Most cases exhibited infiltrative borders (71.1%). 30.4% had positive surgical margins. PNI was the only histological factor significantly associated with an increased risk of recurrence (*P* = .036).

**Table 3:** Pathologic Findings in Stratified by Recurrence

	Total	Recurrence	No recurrence	*P*‐value
Keratinizing	46	11 (23.9%)	35 (76.1%)	
<20%	18 (39.1%)	3 (27.3%)	15 (42.9%)	.36
20%‐50%	23 (50.0%)	8 (72.7%)	15 (42.9%)	.084
>50%	5 (10.9%)	0	5 (14.3%)	.18
Pattern of involvement				
Diffuse sheet like	12 (26.1%)	3 (27.3%)	9 (25.7%)	.92
Diffuse single cell/cord like	8 (17.4%)	2 (18.2%)	6 (17.1%)	.94
Sarcomatoid features	4 (8.7%)	2 (18.2%)	2 (5.7%)	.20
Pseudoglandular/acantolytic	29 (63.0%)	7 (63.6%)	22 (62.9%)	.96
Papillomatous	3 (6.5%)	1 (9.1%)	2 (5.7%)	.69
Giant cell	4 (8.7%)	0	4 (11.4%)	.24
Nest‐like	6 (13.0%)	1 (9.1%)	5 (14.3%)	.66
Border				.18
Infiltrative	33 (71.7%)	9 (81.8%)	24 (68.6%)	
Pushing	12 (26.1%)	1 (9.1%)	11 (31.4%)	
Perineural invasion+	25 (54.3%)	9 (81.8%)	16 (45.7%)	.036
Large nerve PNI (> 1 mm)	7 (15.2%)	2 (18.2%)	5 (14.3%)	.75
Infiltration of vessel wall+	14 (30.4%)	4 (36.4%)	10 (28.6%)	.62
Tumor embolus	17 (37.0%)	3 (27.3%)	14 (40.0%)	.45
Perivascular	9 (19.6%)	2 (18.2%)	7 (20.0%)	.89
Tumor‐related inflammation				
Lymphocyte	39 (84.8%)	8 (72.7%)	31 (88.6%)	.20
PMN	14 (30.4%)	1 (9.1%)	13 (37.1%)	.078
Eosinophil	6 (13.0%)	0	6 (17.1%)	.14
Necrosis+	18 (39.1%)	4 (36.4%)	14 (40.0%)	.83
Node parenchyma	13 (28.2%)	2 (18.2%)	11 (31.4%)	.39
Margins (positive)	14 (30.4%)	3 (27.3%)	11 (31.4%)	.79
WPOI 4/5	20 (43.5%)	5 (45.4%)	15 (42.9%)	.88

Abbreviations: PMN, polymorphonuclear leukocyte; PNI, perineural invasion; WPOI, worst pattern of invasion.

When comparing pathologic characteristics of the 2 WPOI groups, there were no other significant pathologic differences (Table 4).

**Table 4:** Pathologic Findings in Patients Stratified by WPOI

	Total	WPOI 1‐3	WPOI 4‐5	*P*‐value
Keratinizing	46	26 (56.5%)	20 (43.5%)	
<20%	18 (39.1%)	8 (30.7%)	10 (50.0%)	.19
20%‐50%	23 (50.0%)	14 (53.8%)	9 (45.0%)	.55
>50%	5 (10.9%)	4 (15.4%)	1 (5.0%)	.26
Pattern of involvement				
Diffuse sheet like	12 (26.1%)	6 (23.1%)	6 (30.0%)	.60
Diffuse single cell/cord like	8 (17.4%)	4 (15.4%)	4 (20.0%)	.68
Sarcomatoid features	4 (8.7%)	1 (3.8%)	3 (15.0%)	.18
Pseudoglandular/acantolytic	29 (63.0%)	16 (61.5%)	13 (65.0%)	.81
Papillomatous	3 (6.5%)	3 (11.5%)	0	.12
Giant cell	4 (8.7%)	2 (7.7%)	2 (10.0%)	.78
Nest‐like	6 (13.0%)	5 (19.2%)	1 (5.0%)	.16
Perineural invasion+	25 (54.3%)	12 (46.1%)	13 (65.0%)	.20
Large nerve PNI (>1 mm)	7 (15.2%)	3 (11.5%)	4 (20.0%)	.43
Infiltration of vessel wall+	14 (30.4%)	7 (26.9%)	7 (35.0%)	.56
Tumor embolus	17 (37.0%)	7 (26.9%)	10 (50.0%)	.11
Perivascular	9 (19.6%)	7 (26.9%)	2 (10.0%)	.15
Tumor‐related inflammation				
Lymphocyte	39 (84.8%)	23 (88.5%)	16 (80.0%)	.43
PMN	14 (30.4%)	7 (26.9%)	7 (35.0%)	.56
Eosinophil	6 (13.0%)	5 (19.2%)	1 (5.0%)	.16
Necrosis+	18 (39.1%)	11 (42.3%)	7 (35.0%)	.61
Node parenchyma	13 (28.3%)	9 (34.6%)	4 (20.0%)	.28
Margins (positive)	14 (30.4%)	6 (23.1%)	8 (40.0%)	.22

Abbreviations: PMN, polymorphonuclear leukocyte; PNI, perineural invasion; WPOI, worst pattern of invasion.

Additionally, univariate logistic regression showed that patients with PNI were more likely to have recurrence within 2 years than those who did not (OR 8.33, *P* = .023) (Table 5).

**Table 5:** Univariate Logistic Regression Showing the Odds Ratio for Recurrence at 2 Years

	OR with 95% CI	*P*‐value
WPOI 4/5	1.14 (0.25‐5.22)	.86
+PNI	8.33 (1.34‐51.67)	**.023**
Infiltrative border (vs pushing)	3.33 (0.32‐34.83)	.32
Infiltration of vessel wall	4.0 (0.58‐27‐41)	.16
Presence of node parenchyma	0.44 (0.07‐2.79)	.38
Lymph node positive	0.56 (0.11‐2.90)	.49
+margins	0.25 (0.04‐1.71)	.16

Abbreviations: CI, confidence interval; OR, odds ratio; PNI, perineural invasion; WPOI, worst pattern of invasion.

## Discussion

Metastases to the parotid and parotid‐area lymph nodes have been shown to occur in 1% to 3% of patients with cSCC of the head and neck.^6^ Albeit rare, prognosis is poor for this subset of patients. Horakova et al report a 3‐year survival rate for cSCC with metastasis to the parotid gland as only 48%.^15^ For this reason, having a strong understanding of the pathologic factors that contribute to poor prognosis in this subset of patients is critical for treatment planning.

Our study is the first to investigate histopathologic factors such as WPOI in parotid metastases from cSCC of the head and neck region. We specifically focused on WPOI, a commonly accepted poor prognosticator in oral cavity malignancies. In oral mucosal head and neck SCC, WPOI has been shown to have a higher risk of nodal metastasis and poor outcomes.^7^, ^16^ Cutaneous SCC shares numerous histopathologic characteristics and morphological risk factors to oral mucosal SCC. However, the utility of WPOI in cSCC is more controversial. Agar et al investigated WPOI grade in the cSCC and found WPOI grade to be an independent risk factor for nodal metastasis.^8^ To our knowledge, there has yet to be a study investigating WPOI within a metastatic focus of cutaneous SCC, specifically in the parotid gland.

Our cohort of patients posed a unique challenge as the primary tumor information was not available for analysis. It is not uncommon for patients to present to tertiary care centers after having received prior care at other institutions. Consequently, the tertiary care team often does not have all the pathologic information from the prior cSCC in these patients. We hypothesized that the biological nature of the tumors, in terms of their invasive potential and ability to recur and metastasize, would be reflected in their metastatic sites. Because of the histopathological information that was available for these patients, we speculated that this snapshot in the timeline of the patient's disease course could provide useful clinical information in terms of future management and follow up.

Morphological assessment of metastatic sites as a prognostic factor, while not well studied in head and neck cancers, has been studied in several other sites. Specifically, histological growth patterns (HGPs) of liver metastases from colorectal cancers have been highlighted as an important prognostic factor in these cancers. Three distinct patterns have been reported: desmoplastic HGP, pushing HGP, and replacement HGP. It is hypothesized that the desmoplastic HGP could be a good prognostic marker for colorectal cancer with liver metastasis.^17^ For this reason, we sought out to better understand the HGPs of lymph node metastases of cSCC in the head and neck. With a better understanding of these pathologic characteristics, a uniform approach to understanding infiltrative patterns within lymph node metastases could be developed.

Embryologically, lymph node and parotid gland development occur simultaneously, resulting in the presence of intra‐parotid lymph nodes.^18^ The lymph node capsule of intra‐parotid lymph nodes often is less well‐defined compared to a conventional lymph node.^19^ Because of the less‐defined capsule, ENE cannot be assessed in these cases. Additionally, these tumors were so large and infiltrative that there was minimal normal lymph node architecture left behind. For this reason, a surrogate histopathologic factor for ENE, WPOI, was investigated in this subset of patients. As discussed before, WPOI is a known poor prognosticator in oral cavity mucosal SCC. WPOI 5 specifically is defined by satellite lesions away from the main tumor which could theoretically be considered more aggressive than ENE, which is defined by carcinomatous tissue extending beyond the lymph node capsule. The comparison of the two within these metastatic deposits is critical in determining their prognostic value.

In our study, parotid metastasis with a higher WPOI grade was associated with a prior need for excision with adjuvant treatment suggesting a more aggressive or incompletely excised primary tumor. Additionally, those found to have a high WPOI were associated with an increased rate of chemotherapy and radiation treatment after parotidectomy with or without neck dissection. Despite the more aggressive pathologic phenotype, WPOI grade did not have a significant effect on recurrence rate in these tumors. Most likely, this could be attributed to most patients with a higher WPOI receiving a more aggressive adjuvant therapy and treatment regimen. These patients did seem to respond to this with a survival statistically equal to the lower WPOI patients.

Additionally, in our small study, patients with PNI were more likely to have recurrence, suggesting that PNI is a more important factor to consider when determining prognosis in these patients even though they are already considered metastatic.

On univariate logistic regression, PNI continued to be a statistically significant risk factor for recurrence. However, a larger study is needed to confirm the utility of PNI in this unique subset of patients.

Current National Comprehensive Cancer Network (NCCN) guidelines do not routinely recommend a parotidectomy and neck dissection on a head and neck SCC without evidence of nodal disease. With specific regards to parotid SCC, there are several studies that recommend a neck dissection with parotidectomy for SCC of the parotid gland because these tumors are considered high‐grade.^20^, ^21^, ^22^ The type of surgical management, however, is dependent on the location of the primary tumor, and surgical management can be largely surgeon dependent. Furthermore, sentinel lymph node biopsy (SNLB) can be considered in patients with a high‐risk or very high‐risk cSCC.

Limitations to the study include the small sample size and incomplete data regarding primary tumors. Data were collected in a retrospective manner, and follow‐up data were not consistent. Our patient population was also complicated by a high incidence of recurrence prior to metastasis to the parotid. These lesions are generally primarily treated by dermatologists, and pathology reports are frequently developed by dermatologists and dermatopathologists. The other major limitation of this study is the absence of molecular data and programmed death‐ligand 1 (PD‐L1) status of these tumors. In future studies, it would be beneficial to gather histopathologic information from both the primary and metastatic tumors in a large patient cohort from multiple institutions with reliable 5‐year follow‐up with incorporation of molecular and PDL1 immunostaining data.

In conclusion, this study is the first, to our knowledge, to examine WPOI and other histopathologic factors in cSCC with parotid gland metastasis. Our findings support the hypothesis that prognostic features identified at metastatic sites—such as PNI and WPOI patterns—should be incorporated into pathology reports to guide therapeutic decision‐making. As advances in targeted and immunotherapies expand in adjuvant and neoadjuvant settings, integrating morphologic and molecular characterization of these tumors will be essential. A clearer understanding of the aggressive histopathologic profile of cSCC with parotid metastases may enable clinicians to make more informed choices for managing these high‐risk malignancies.

## Author Contributions

**Mayuri Yasuda**, design, conduct, analysis, presentation; **Aayushma Regmi**, conduct; **Maitri Mehta**, conduct; **Cullen M. Lilley**, conduct; **Eric Thorpe**, conduct, analysis; **Swati Mehrotra**, design, conduct; **Andrea Ziegler**, design, conduct, analysis; **Vijayalakshmi Ananthanarayanan**, design, conduct, analysis.

## Disclosures

### Competing interests

None.

### Funding source

None.
